# Understanding the disparity of educational attainment in Northern Ireland: The role of socio-demographic and school-level factors on GCSE attainment.

**DOI:** 10.23889/ijpds.v8i2.2197

**Published:** 2023-09-14

**Authors:** Erin Early, Sarah Miller, Laura Dunne, John Moriarty

**Affiliations:** 1 Queen's University Belfast, Belfast, United Kingdom

## Objectives

This study examined the individual and collective impacts of socio-demographics and school-level factors on GCSE outcomes in Northern Ireland, using linked administrative data. A pupil’s sex, religious affiliation and socio-economic background (measured by eight measures) were examined, along with parental socio-economic background, attended school type (grammar/non-grammar) and school management structure.

## Method

This study used the first linked administrative dataset for education in Northern Ireland. The dataset linked the 2011 household Census, School Leavers Survey (2010-2014) and School Census (2010-2014) for the first time. Data were provided for three pupil cohorts who completed their GCSE examinations in consecutive academic years (2010/2011 – 2012/2013).

The study conducted multilevel models to understand the nested effects of pupil-, household- and school-level factors on GCSE attainment outcomes. Interaction models were also executed to examine the multiplicative effects of a pupil’s sex, religious affiliation and socio-economic background on their educational attainment.

## Results

The findings of this study highlight that the impact of socio-economic status is multidimensional, with some measures having a greater impact on GCSE attainment than others. For example, a mother’s education qualifications had the largest impact of socio-economic measures included in the multilevel models. The analysis also found that Free School Meal Eligibility remains an important predictor of attainment outcomes. When considering pupils’ sex, females had higher GCSE attainment scores than males. However, religious affiliation had a varied influence on GCSE outcomes, indicating the need for a more nuanced approach when considering this factor. The importance of interaction terms to gain an in-depth understanding of the multiplicative effect of factors on attainment outcomes was also highlighted in the analysis.

## Conclusion

Through the use of linked administrative data, this study highlights the hierarchy of socio-economic effects on GCSE attainment outcomes in Northern Ireland. It also highlights the importance of collectively considering the factors that make up a pupil’s demographic profile to garner a holistic understanding of attainment trends in Northern Ireland.

